# Assembly of the poorly differentiated *Verasper variegatus* W chromosome by different sequencing technologies

**DOI:** 10.1038/s41597-023-02790-z

**Published:** 2023-12-13

**Authors:** Xi-wen Xu, Pengchuan Sun, Chengbin Gao, Weiwei Zheng, Songlin Chen

**Affiliations:** 1 State Key Laboratory of Mariculture Biobreeding and Sustainable Goods, Yellow Sea Fisheries Research Institute, Chinese Academy of Fishery Sciences, Qingdao, 266071 China; 2 Laboratory for Marine Fisheries Science and Food Production Processes, Laoshan Laboratory, Qingdao, 266237 China; 3 Key Laboratory for Bio-resources and Eco-environment & Sichuan Zoige Alpine Wetland Ecosystem National Observation and Research Station, College of Life Sciences, Sichuan University, Chengdu, 610065 China

**Keywords:** Genetics, Genome

## Abstract

The assembly of W and Y chromosomes poses significant challenges in vertebrate genome sequencing and assembly. Here, we successfully assembled the W chromosome of *Verasper variegatus* with a length of 20.48 Mb by combining population and PacBio HiFi sequencing data. It was identified as a young sex chromosome and showed signs of expansion in repetitive sequences. The major component of the expansion was Ty3/Gypsy. The ancestral Osteichthyes karyotype consists of 24 protochromosomes. The sex chromosomes in four Pleuronectiformes species derived from a pair of homologous protochromosomes resulting from a whole-genome duplication event in teleost fish, yet with different sex-determination systems. *V. variegatus* and *Cynoglossus semilaevis* adhere to the ZZ/ZW system, while *Hippoglossus stenolepis* and *H. hippoglossus* follow the XX/XY system. Interestingly, *V. variegatus* and *H. hippoglossus* derived from one protochromosome, while *C. semilaevis* and *H. stenolepis* derived from another protochromosome. Our study provides valuable insights into the evolution of sex chromosomes in flatfish and sheds light on the important role of whole-genome duplication in shaping the evolution of sex chromosomes.

## Background & Summary

Although the assembly of W and Y chromosomes presents one of the most daunting challenges in sequencing and assembling vertebrate genomes, it remains a crucial area of research^1^. The analysis of W and Y chromosomes can provide valuable insights into the specific evolutionary trajectory for females and males^2^. Sex chromosomes evolved from a pair of autosomes^3,4^, and with the accumulation of antagonistic sites around the sex determining gene, a recombination inhibition region was formed and gradually expanded, eventually leading to the difference between X and Y (or Z and W) chromosomes^5,6^. Although stable sex-determination systems controlling male and female differentiation were formed in a variety of vertebrate taxa, such as mammals and birds^7–9^, sex chromosomes have evolved independently many times throughout the tree of life, resulting in the existence of a diversity of sex chromosomes, especially in fish and amphibians^10–12^. Bony fish are the most species-rich group of vertebrates, containing nearly 30,000 species, accounting for approximately 98% of ray-finned fish and 50% of vertebrate species^13^. However, less than 10% of fish sex chromosomes are heteromorphic in karyotype, and most of them are young sex chromosomes, that is, in the early stage of differentiation^14^. Studying young sex chromosomes provides an opportunity to study the initiation process of sex chromosome recombination inhibition^15^. However, the low degree of differentiation and high sequence similarity between young sex chromosomes is bringing some challenges to chromosome phasing and assembly^16^. The development of sequencing technologies, especially high-precision PacBio HiFi sequencing, has brought opportunities for genome haplotype assembly^17^.

*V. variegatus*, *H. hippoglossus* and *H. stenolepis* belong to the family Pleuronectidae, and they have evolved different sex-determination systems. The sex-determination system of the *H. stenolepis* and *V. variegatus* is ZZ/ZW^18,19^, and the sex-determination system of *H. hippoglossus* is XX/XY^20^. The sex-determining genes of *H. hippoglossus* and *H. stenolepis* are also different. To date, there has been little research on the evolution of sex chromosomes and the transformation mechanism of the sex determination system in Pleuronectidae. Here, by combining population and PacBio HiFi sequencing data, we successfully assembled the W chromosome of *V. variegatus*, and preliminarily discussed the origin of the sex chromosome of *V. variegatus* through collinearity analysis, which provided rich resources for follow-up research on the mechanism of sex chromosome evolution in flatfish.

## Materials and Methods

### *V. variegatus* samples and sequencing

For PacBio CLR and HiFi sequencing, we used a female adult *V. variegatus* for DNA sequencing. DNA extraction was performed using the SDS-based method, the ground tissue cells were lysed with hot SDS, and high concentrations of KAc were added to remove proteins and polysaccharide impurities by incubating at 0 °C. Finally, precipitation was performed using ethanol or isopropanol. After fragmentation, the BluePippin Size Selection system (Sage Science) was used to screen DNA fragments of approximately 20 kb. Then, PacBio CLR and HiFi libraries were constructed according to the PacBio standard library construction process and sequenced using the PacBio SEQUEL platform. We obtained 53.71 Gb of female V. variegatus PacBio continuous long read (CLR) sequencing data.

For Illumina sequencing, after DNA extraction and fragmentation from 18 female and 17 male fish, 300 bp pair-end Illumina libraries were constructed according to the Illumina standard library construction process and then sequenced using the Illumina NovaSeq. 6000 platform. We obtained 36.38 Gb Illumina sequencing data.

### Genome assembly

To obtain high-quality female *V. variegatus* genome sequences, we used the process shown in Supplementary Fig. 1 to assemble the genome. Canu v1.8^21^ (-correct) was first used to correct raw PacBio data. Then, we used Flye v2.6^22^ (–pacbio-corr) to obtain a draft genome with the corrected PacBio data. The draft genome may have partially redundant sequences, so, we used purge_dups v1.0.0^23^ to remove redundant sequences. Then we performed two rounds of genome polishing using PacBio and resequencing data. The detailed process for polishing the genome was as follows: (1) the PacBio data were aligned to the genome by pbmm2 (SMRT Link v8.0) with default parameters, and then gcpp (SMRT Link v8.0) with default parameters was used to polish the genome. (2) the resequencing data were aligned to the genome by bwa v0.7.17^24^ with default parameters, and pilon v1.23^25^ with default parameters was used to polish the genome. After two rounds of genome polishing with software gcpp and pilon. Finally, combining PacBio and Illumina data, we obtained 543.90 Mb female *V. variegatus* contigs, of which the contig N50 was 17.26 Mb (Supplementary Table 1).

To obtain the chromosome-level genome, we first aligned Hi-C data (SRP263299) to contigs using juicer v1.6.2^26^ with default parameters and then anchored contigs to chromosomes using 3D-DNA^27^ (-r 0). 99.89% of female sequences were anchored to 23 pseudochromosomes. Both female and male fish have 23 mounted chromosomes, which means no additional W chromosome was assembled in the female genome. Then we used nucmer to compare female and male genomes and found that the genome identity was more than 99.7%. It may be that the Z and W chromosomes may have a low degree of differentiation and the PacBio CLR data with a relatively high error rate cannot be used for the correct phase of Z and W chromosomes, causing the female Z and W chromosomes to mix-assemble into one chromosome.

### W chromosome assembly

To obtain the *V. variegatus* W chromosome, we assembled it by combining PacBio HiFi sequencing data and whole genome resequencing data (Supplementary Fig. 1B). We first used the whole genome resequencing data to obtain female specific kmers according to the following steps: (1) The 17-kmer dataset of each individual was obtained separately using meryl count^28^. (2) For individual female 17-kmer datasets, kmers with frequency less than 5 were removed using meryl greater-than 5. (3) Individual 17-kmer datasets of 18 female fish were combined into a total datasest using meryl union-sum, considering only the k-mers that were present in all 18 individuals. (4) All kmer data of 18 male fish were combined into a total set using meryl union-sum. (5) Select kmers that exist in all female fish but do not exist in any male fish were identified as specific kmers using meryl difference. After obtaining female-specific kmers, we used meryl-lookup to screen PacBio HiFi sequencing reads containing female-specific kmers. A total of 573,403 female-specific kmers were identified by comparing the resequencing data of female and male fish. Subsequently, by leveraging female-specific kmers, we were able to filter out female-specific data from PacBio HiFi and Hi-C sequencing data, enabling us to conduct a more in-depth analysis of the genetic differences unique to female flatfish. In total, 515.87 Mb female-specific PacBio HiFi data and 189.05 Mb of Hi-C data were screened out. These HiFi data were used to assemble W candidate sequences using Flye with default parameters, a total of 20.48 Mb genome sequence with a contig N50 of 5.16 Mb was obtained. Then we used juicer and 3D-DNA to anchor contigs to chromosomes using the female-specific Hi-C data.

**Fig. 1:**
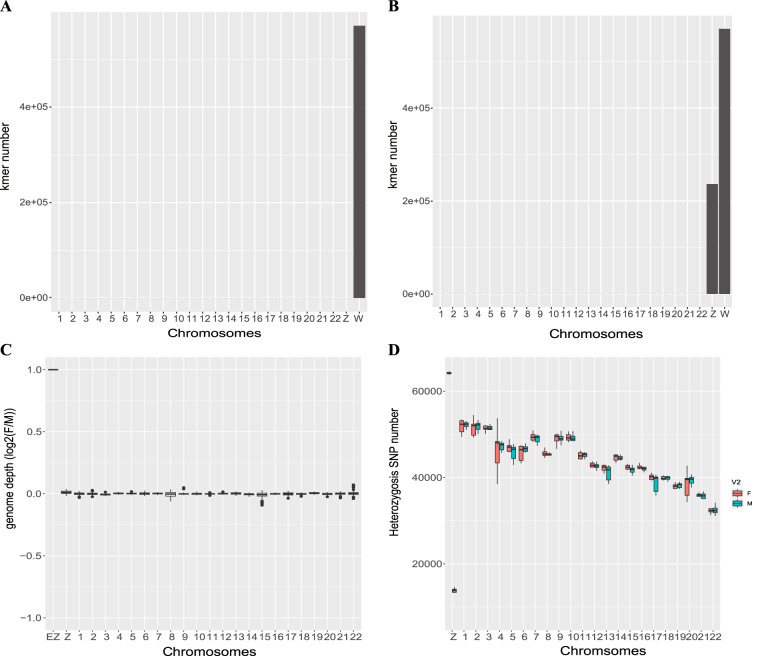
Analysis of basic characteristics of W chromosome. (**A**) Distribution of female specific Kmer in male genome, chromosome 1 to 23 belonged to male genome, chromosome W belonged to female genome assembled by combing population and PacBio HiFi data. (**B**) Distribution of female specific Kmer in female genome, chromosome 1 to 23 belonged to female genome assembled by PacBio CLR data, chromosome W was assembled by combing population and PacBio HiFi data. (**C**) Expected genome depth in females relative to males (log2(M/F)), EZ was the expected genome depth in males relative to females (log2(M/F) ≈ 1), chromosome 1 to 23 belonged to male genome. (**D**) Distribution of heterozygous SNPs in females and males.

After obtaining the W chromosome, we used the W chromosome information to identify Z chromosomes in the female and male genomes^29^, respectively. After downloading the male *V. variegatus* genome from the NCBI GenBank database (GCA_013332515.1), we used MashMap v2.0^30^ (–noSplit) to map the W chromosome to the male genome and found more than 95% sequence identity between chromosome LG21 and the W chromosome. Additionally, we found that the sequence identity of chromosomes 18 and W in the female assembled genome was more than 96%. Therefore, chromosomes LG21 and chromosome 18 were candidate Z chromosomes for the male and female genomes, respectively. Then, we examined the distribution of female-specific kmers in the female and male assembled genomes using meryl-lookup. In male genomes, almost all female-specific kmers were distributed only on the W chromosome (Fig. 1A), while in female genome sequences, female-specific kmers were distributed abundantly on the Z chromosomes in addition to the W chromosome (Fig. 1B). The above results showed that candidate Z chromosomes in the female genome have mixed W chromosome sequences. Then, we used nucmer^31^ to align the W and Z chromosomes, and dnadiff to show the sequence identity between the W and Z chromosomes was more than 96%. Furthermore, we used bwa to align 18 male and 18 female resequencing data to the genome of male *V. variegatus*. If the W and Z chromosomes of the *V. variegatus* genome were fully differentiated, the depth of the Z chromosome in the male sequencing data should be twice that of the female sequencing data (${\rm{EZ}}{:\log }_{2}\frac{{\rm{M}}}{{\rm{F}}}\approx 1$), with the decrease in differentiation degree, the coverage of male fish and female fish tended to be the same (${\log }_{2}\frac{{\rm{M}}}{{\rm{F}}}\approx 0$). We found that the sequencing depth of all chromosomes did not show significant differences (${\log }_{2}\frac{{\rm{M}}}{{\rm{F}}}\approx 0$), that is, there were no significant differences in genome sequences between Z and W chromosomes (Fig. 1C). This indicates that the *V. variegatus* W and Z chromosomes may be homomorphic sex chromosomes. To further analyze the characteristics of *V. variegatus* sex chromosomes, we used freebays^32^ to perform SNP calling on 18 female and 18 male fish found that except for the Z chromosome, male and female fish have a similar number of heterozygous SNPs. The number of heterozygous SNPs on the Z chromosome in females is between 63875–64986, with an average of 64201, while the number of heterozygous SNPs on the Z chromosome in males is between 10585–14598, with an average of 13598 (Fig. 1D), in other words the number of heterozygous SNPs on the Z chromosome was significantly higher in females than in males (Fig. 1D), indicating that Z and W chromosomes have started to differentiate. Previous studies have found that homomorphic sex chromosomes could accumulate SNPs before the sex chromosome decays, and differences in heterozygosity between males and females could be detected even if the sex chromosomes do not show differences in coverage^33^. Therefore, the *V. variegatus* W chromosome is a nascent sex chromosome, that could provide valuable resources for studying early sex chromosome differentiation and understanding the initial process of recombination inhibition.

We used EDTA v2.00^34^ (–sensitive 1–anno 1) to identify repeated sequences. The content of repeated sequences on the W chromosome was 1.75 Mb, which was higher than that on the Z chromosome (1.44 Mb), and the expanded repeated sequences on the W chromosome were mainly Ty3/Gypsy (Supplementary Table 2). Compared with the degenerated and smaller W/Y chromosomes of mammals and birds, the W/Y chromosomes of many fish are nascent sex chromosomes and larger than the Z/Y chromosomes^2,35,36^. Repeated sequences played an important role in the formation of sex determining regions of nascent W/Y chromosomes and could lead to an increase in chromosome size by accumulating repetitive sequences at the early stage of differentiation^37–40^. It has been proven that the transposable element Ty3/Gypsy is closely related to sex specific and sex related regions, which means that Ty3/Gypsy may play a role in the process of sex chromosome differentiation and the formation of new sex determination related regions^41–44^.

Then we used MAker2^45^ to predict genes in W chromosome. To predict genes, MAker2 used SNAP^46^ and Augustus^47^ for de novo gene prediction and Exonerate^48^ to align non-redundant proteins from flatfish and *V. variegatus* RNA-seq data (SRP263299) to the genome for homologous gene prediction. Finally, EVM^49^ was used to integrate information from de novo and homologous gene predictions to obtain the final gene structure. We annotated the W chromosome gene structure with MAKER2, and 1,033 protein coding genes were annotated.

Finally, we improved the autosomes and Z chromosome of *V. variegatus* using the female and male genome sequences. Briefly, we used genome puzzle master (GPM)^50^, an integrated pipeline for building and editing pseudomolecules from fragmented sequences, to integrate the female and male genome sequences and obtained a 543.54 Mb *V. variegatus* genome sequence, whose contig N50 and scaffold N50 sizes were 22.76 Mb and 24.82 Mb, respectively. Then, Liftoff^51^ (v1.6.1) with default parameters was used to map the annotations of male genome to the newly assembled genome.

### Collinearity analysis of sex chromosomes

**Fig. 2:**
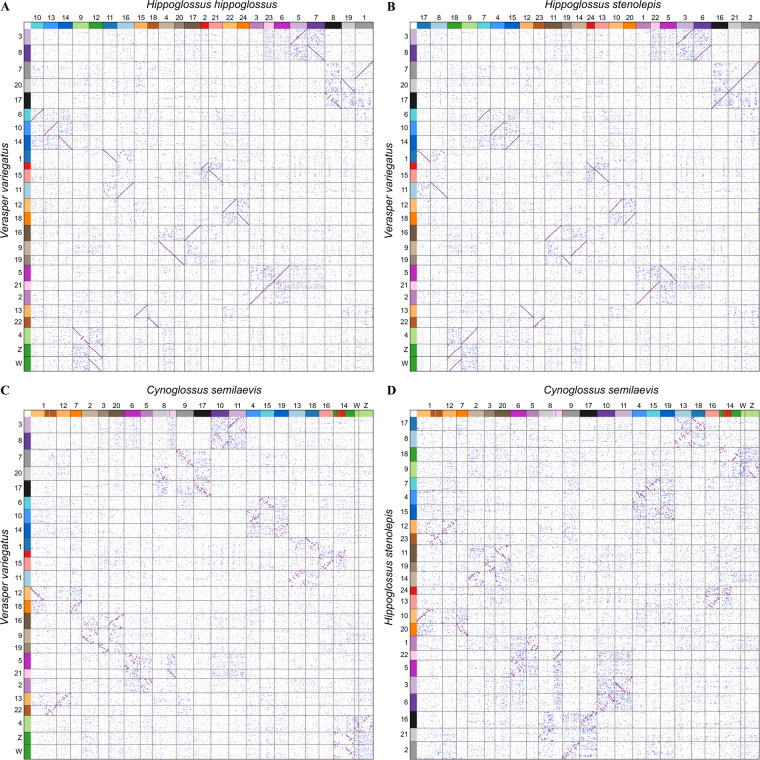
Collinearity analysis of four species of flatfish. (**A**) Collinearity analysis between *V. variegatus* and *H. hippoglossus*. (**B**) Collinearity analysis between *V. variegatus* and *H. stenolepis*. (**C**) Collinearity analysis between *V. variegatus* and *C. semilaevis*. (**D**) Collinearity analysis between *C. semilaevis* and *H. stenolepis*.

Seven teleosts genomes, including *Mastacembelus armatus* from Synbranchiformes, *Gasterosteus aculeatus* from Perciformes, *Poecilia reticulata* from Cyprinodontiformes, and *C. semilaevis*, *V. variegatus*, *H. hippoglossus* and *H. stenolepis* from Pleuronectiformes, each with specific sex chromosome information, were selected for collinearity analysis using WGDI^52^. Through genome collinearity analysis, the sex chromosomes (Z and W) of *V. variegatus* had the best collinearity relationship with the sex chromosome (chromosome 12) of *H. hippoglossus*, which indicates that they came from the same paleochromosome. From the dotplot, we also found that the sex chromosomes of *V. variegatus* had an ancient collinearity relationship with the autosome (chromosome 9) of *H. hippoglossus* (Fig. 2A). The comparative analysis of the collinearity between *V. variegatus* and *H. stenolepis* showed that the sex chromosomes (Z and W) of *V. variegatus* had the best collinear relationship with the autosome (chromosome 18) of *H. stenolepis*, and had an ancient collinearity relationship with the sex chromosome (chromosome 9) of *H. stenolepis* (Fig. 2B). Furthermore, we compared the collinearity between *V. variegatus* and *C. semilaevis*, and found that their collinear relationship was similar to that of *H. stenolepis*, and the Z and W chromosomes of *V. variegatus* had the best collinear relationship with the autosome (chromosome 3) of *C. semilaevis*, but had an ancient collinear relationship with the sex chromosomes (Z and W) of *C. semilaevis* (Fig. 2C). Then we further compared the collinearity between *C. semilaevis* and *H. stenolepis* and found that the sex chromosomes of *C. semilaevis* and *H. stenolepis* had the best collinear relationship, which indicates that they came from the same paleochromosome (Fig. 2D). In addition, by integrating all the dotplot information, we also found that the sex chromosomes of *V. variegatus* and *H. hippoglossus* and the sex chromosomes of *C. semilaevis* and *H. stenolepis* had an ancient collinear relationship, respectively. *V. variegatus*, *C. semilaevis*, *H. hippoglossus* and *H. stenolepis* did not experience an additional whole genome duplication event after the common whole genome duplication event of teleost fish^53,54^. In other words, the paleochromosomes of *V. variegatus* and *H. hippoglossus* and the paleochromosomes of *C. semilaevis* and *H. stenolepis* came from the same ancient chromosome. It also provides the evidence for the emergence of young sex chromosomes from autosomes and the exceptional evolutionary instability of sex chromosomes in fish^55^.

**Fig. 3:**
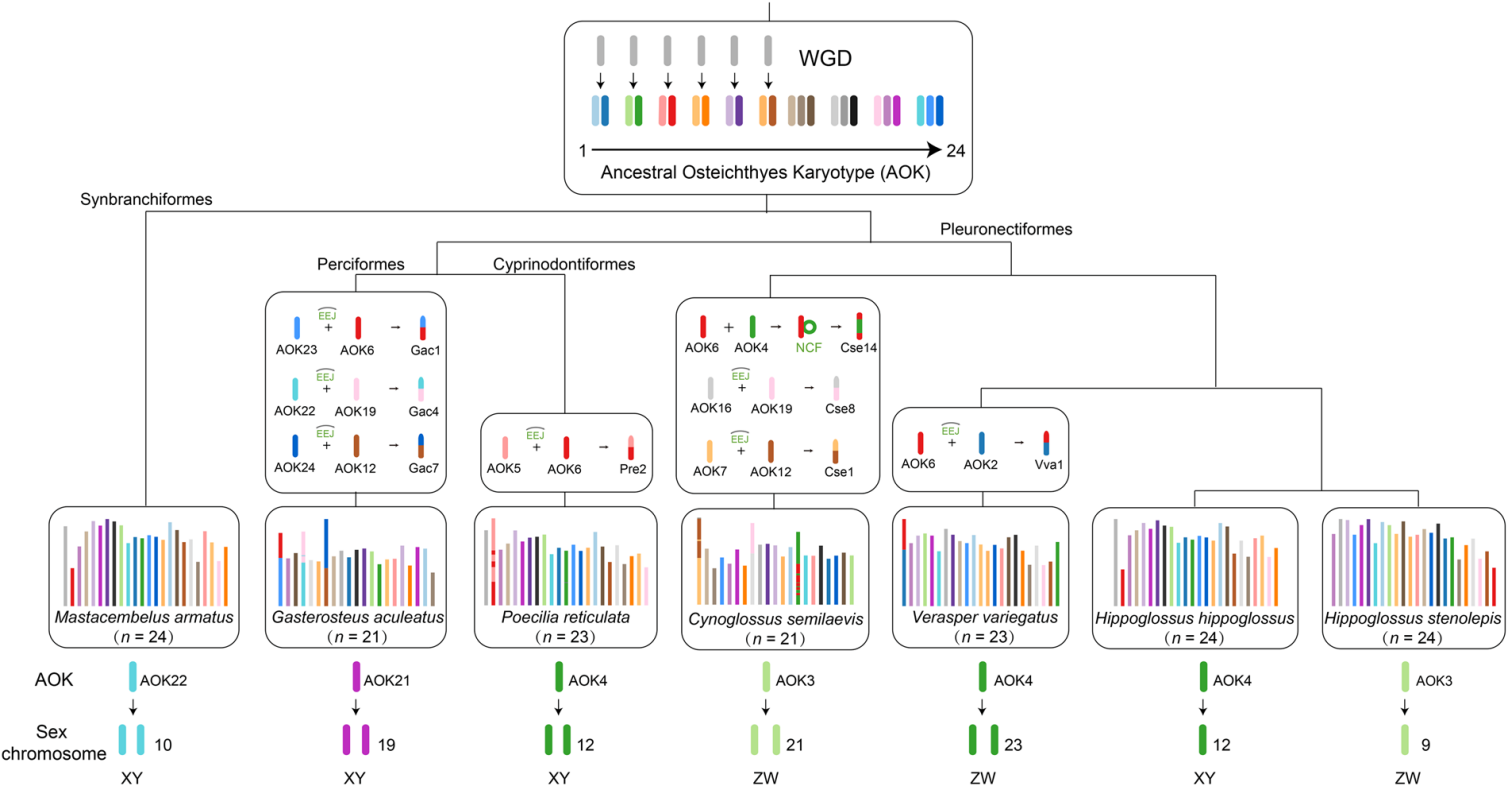
The evolution and origin analyses of Ancestral Osteichthyes Karyotype (AOK) in seven teleost fishes. (*M. armatus, G. aculeatus, P. reticulata, C. semilaevis, V. variegatus, H. hippoglossus and H. stenolepis*) from 4 orders (Synbranchiformes, Perciformes, Cyprinodontiformes and Pleuronectiformes).

We applied the workflow (https://github.com/SunPengChuan/wgdi-example/blob/main/Karyotype_Evolution.md) to identify protochromosomes and resulted in an ancestral Osteichthyes karyotype (AOK) of 24 protochromosomes (Fig. 3). The sex chromosomes of five genomes (four from Pleuronectiformes and one from Cyprinodontiformes) were derived from a pair of homologous chromosomes (AOK3 and AOK4), except for *M. armatus* from AOK22 and *G. aculeatus* from AOK21. Meanwhile, *H. hippoglossus* and *H. stenolepis* both have 24 chromosomes without any fusion or fission, but possess different sex determination systems (XX/XY and ZZ/ZW, respectively), and the origins of the sex chromosomes are also different in these two species (the former from AOK4 and the latter from AOK3). Coincidentally, AOK4 occurred a fusion event (NCF) in *C. semilaevis*. These results will provide valuable resources for follow-up research on the mechanism of sex chromosome evolution and the transformation mechanism of sex determination system of flatfish.

## Data Records

The genome assembly related data was submitted to the NCBI SRA database with accession number SRP396161^56^. The genome sequence has been submitted to the GenBank database, GenBank assembly accession: GCA_026259375.1^57^. Gene structure and transposition factor annotation files have been deposited in Figshare^58^.

## Technical Validation

To assess genome assembly quality, we evaluated it from different aspects. We first used BUSCO 5.3.2^59^ (-l actinopterygii_odb10) to assess the assembly completeness and found that the genome completeness reached 98.1%, and only 1.4% of BUSCO groups were not found in the genome. We then used several different methods to assess genome assembly quality. We assessed genome assembly quality based on the kmer strategy, to estimate the assembly accuracy to be 99.98%. In addition, we used freebays to search for homozygous SNPs that may be assembled base errors and found 7,215 homozygous SNPs, indicating that the base accuracy rate was as high as 99.998%. Finally, we used Illumina sequencing data to assess the sequence support of the genome. After aligning the Illumina sequence data back to the genome using bwa v0.7.17 with default parameters, we then used SAMtools^60^ flagstat to calculate basic statistics and found that 99.48% of the reads could be mapped back to the genome, of which 98.14% of the reads had the correct pairing orientation. We used SAMtools depth to count genome base coverage and found that 99.93% of bases were covered by at least 5 reads. Overall, the genome had good integrity and assembly quality.

### Supplementary information

Supplementary figures and tables

## Data Availability

The data analysis methods, software and associated parameters used in present study are described in the section of Methods. If no detail parameters were described for software used in this study, default parameters were employed. No custom scripts were generated in this work.
