# Supplementary material for: Assembly of the poorly differentiated Verasper variegatus W chromosome by different sequencing technologies
Source: Sci Data. 2023 Dec 13;10:893. doi: 10.1038/s41597-023-02790-z (PMC10719390; doi:10.1038/s41597-023-02790-z)
Supplement: Supplementary file 1 — Supplementary figures and tables [file 41597_2023_2790_MOESM1_ESM.docx]

**Assembly of the poorly differentiated *Verasper variegatus* W chromosome by different sequencing technologies**

Xi-wen Xu^1,2^, Pengchuan Sun^3^, Chengbin Gao^1^, Weiwei Zheng^1^,Songlin Chen^1,2*^

^1^ State Key Laboratory of Mariculture Biobreeding and Sustainable Goods, Yellow Sea Fisheries Research Institute, Chinese Academy of Fishery Sciences, Qingdao 266071, China.

^2^ Laboratory for Marine Fisheries Science and Food Production Processes, Laoshan Laboratory, Qingdao 266237, China

^3^ Key Laboratory for Bio-resources and Eco-environment & Sichuan Zoige Alpine Wetland Ecosystem National Observation and Research Station, College of Life Sciences, Sichuan University, Chengdu, 610065, China.

*Correspondence and requests for materials should be addressed to S.L.C. (chensl@ysfri.ac.cn)

**This file includes:**

**Supplementary Figures 1**

**Supplementary Tables 1-2**


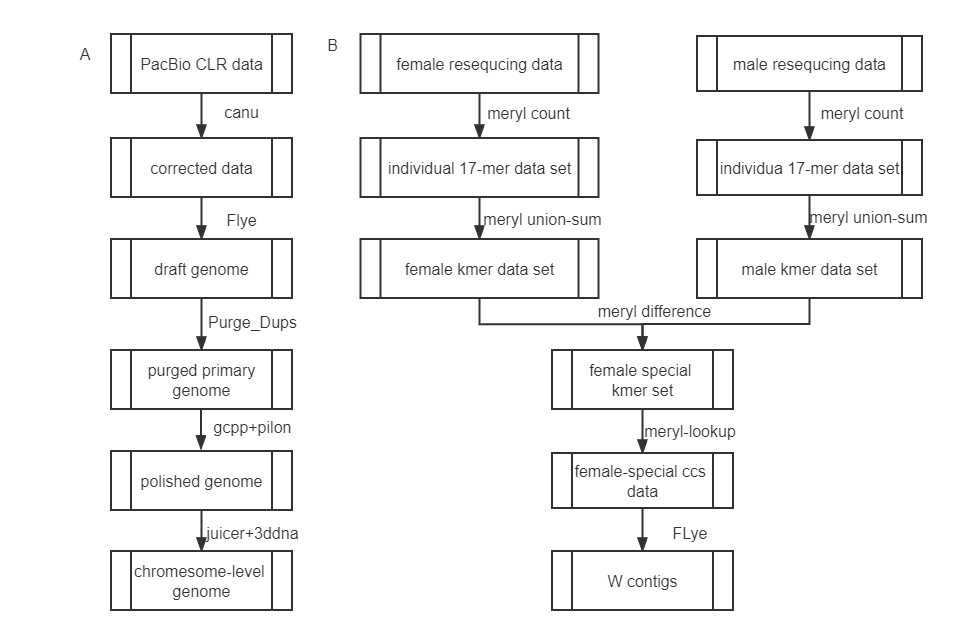


**Supplementary Figure** **1. Assembly flow chart** (A) Flow Chart of Female Genome Assembly (B) Flow Chart of W Chromosome Assembly

**Supplementary Tables**

**Supplementary Table 1:** Basic assembly information statistics

|  | Contigs | Scaffolds |
| --- | --- | --- |
| Toltal length (Mb) | 564.00 | 564.01 |
| Sequence count | 79 | 34 |
| Longest sequence (Mb) | 30.68 | 31.79 |
| N50 | 22.76 | 24.82 |
| L50 | 10 | 10 |
| N90 | 14.86 | 19.54 |
| L90 | 22 | 20 |

**Supplementary** **Table 2**. Comparative statistic of the Z and W repeat sequences

| Class | | Z | | W | |
| --- | --- | --- | --- | --- | --- |
|  |  | Length (bp) | % in Genome | Length (bp) | % in Genome |
| LTR | Gypsy | 44,222 | 0.22 | 353,966 | 1.73 |
|  | unknown | 41,825 | 0.21 | - | - |
| TIR | CACTA | 273,405 | 1.35 | 289,072 | 1.41 |
|  | Mutator | 135,341 | 0.67 | 120,383 | 0.59 |
|  | PIF_Harbinger | 77,904 | 0.39 | 14,149 | 0.07 |
|  | Tc1_Mariner | 4,421 | 0.02 | 8,167 | 0.04 |
|  | hAT | 120,800 | 0.6 | 129,072 | 0.63 |
| nonLTR | LINE_element | 56,067 | 0.28 | 85,140 | 0.42 |
| nonTIR | helitron | 215,244 | 1.07 | 215,786 | 1.05 |
|  | repeat_region | 467,536 | 2.32 | 531,059 | 2.59 |
| Total | | 1,436,765 | 7.11 | 1,746.79 | 8.53 |
